# Repetitive optogenetic stimulation of glutamatergic neurons: An alternative to NMDA treatment for generating locomotor activity in spinalized zebrafish larvae

**DOI:** 10.14814/phy2.14774

**Published:** 2021-03-26

**Authors:** Jacob E. Montgomery, Sarah Wahlstrom‐Helgren, Kayce T. Vanpelt, Mark A. Masino

**Affiliations:** ^1^ Department of Neuroscience University of Minnesota Minneapolis MN USA

**Keywords:** locomotion, optogenetic, spinal cord, zebrafish

## Abstract

*N*‐methyl‐d‐aspartate (NMDA) application has conventionally been used to activate spinal networks to induce locomotion in spinalized animals. We recently described an alternative approach in which application of continuous blue light activates channelrhodopsin‐2 in *vesicular glutamate transporter 2a* (*vglut2a*)‐expressing spinal neurons to produce organized, rhythmic locomotor activity in spinally‐transected larval zebrafish. This technique arguably enhances research validity, because endogenous glutamate is released into existing synapses instead of activating only a subset of glutamatergic (NMDA) receptors with an exogenous compound. Here, we explored the viability of this approach in the context of using it for longer‐term experiments. Fictive swimming was induced through repetitive application of 10‐s blue light stimuli to spinalized preparations for up to 60 min at intervals of 1, 3, or 15 min. Locomotor activity was maintained throughout the experimental timecourse, demonstrating the robustness of the system. Although locomotor bursts remained organized into episodes of activity, the number of bursts elicited during each successive stimulus decreased. This was in contrast to NMDA bath application, in which bursts became less episodically organized while the overall number of bursts remained unchanged. The efficacy of the repetitive optogenetic stimulation paradigm was demonstrated through application of exogenous dopamine, which reversibly decreased the number of bursts produced per stimulus compared with untreated preparations. Finally, increasing the stimulus interval to 15 min lessened, but did not eliminate locomotor fatigue from repetitive activation. Altogether, we established repetitive optogenetic stimulation of *vglut2a*‐expressing neurons as a viable alternative to NMDA application for activation of the zebrafish spinal locomotor network.

## Graphical abstract

Neuronal circuits within the spinal cord have the capacity to produce rhythmic locomotor activity in isolation from the brain. Application of exogenous *N*‐methyl‐d‐aspartate (NMDA) is commonly used to experimentally excite these networks to produce locomotor activity. Here, we demonstrate that repetitive optogenetic activation of spinal vesicular glutamate transporter 2a‐expressing neurons is a viable, and potentially more biologically‐relevant, alternative to NMDA bath application for studying locomotor network modulation.

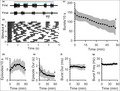

**New and noteworthy**

Neural circuits within the spinal cord have the capacity to produce rhythmic locomotor activity in isolation from the brain. Application of exogenous *N*‐methyl‐d‐aspartate (NMDA) is commonly used to experimentally excite these networks to produce locomotor activity. Here, we demonstrate that repetitive optogenetic activation of spinal *vesicular glutamate transporter 2a*‐expressing neurons is a viable, and potentially more biologically‐relevant, alternative to NMDA bath application for studying locomotor network modulation.

## 1 INTRODUCTION

Complete spinal cord transections lead to an absence of voluntary locomotor function below the injury. In humans this is expressed as paraplegia or tetraplegia, depending on the spinal level of the injury. Spinal central pattern generators (CPGs) retain the capacity to produce coordinated locomotor output in the absence of descending input from the brain (Grillner, 1985). Hence, sufficient stimulation of these networks can induce coordinated rhythmic locomotor output and is being explored as a means to restore volitional locomotion following spinal cord injury (Minassian & Hofstoetter, 2016).

Vertebrate animal models are commonly exploited to interrogate the CPGs that underlie spinally‐produced locomotor activity. Locomotor activity is induced in spinal nervous tissue through application of various excitatory compounds, such as *N*‐methyl‐d‐aspartate (NMDA; Cazalets et al. 1992; Grillner et al. 1981) or electrical stimulation (Iwahara et al. 1992). More recently, the advent of optogenetic technologies has allowed researchers to activate genetically‐specified neuronal cell populations to investigate their roles in producing locomotion.

Activation of spinal glutamatergic (*Vglut2*‐expressing in mouse, *vglut2a*‐expressing in zebrafish) neurons with Channelrhodopsin‐2 (ChR2) is sufficient to drive stereotyped locomotor activity in spinally transected (spinalized) mice (Hägglund et al. 2010) and larval zebrafish (Ljunggren et al. 2014; Wahlstrom‐Helgren et al. 2019). In zebrafish, selective optogenetic activation of only glutamatergic V2a interneurons initiates coordinated fictive swimming activity (Ljunggren et al. 2014). However, it is unclear if V2a activation alone provides sufficient excitation to produce multiple episodes, or bouts, of swimming during a single, continuous stimulus. In comparison, activation of all *vglut2a*‐expressing glutamatergic spinal cell types elicits multiple episodes of coordinated swimming during a single, continuous stimulus (Wahlstrom‐Helgren et al. 2019). V2a interneurons directly synapse onto motor neurons (Kimura et al. 2006; Svara et al. 2018), providing excitatory drive and regulating locomotor frequency (Ampatzis et al. 2014; Eklöf‐Ljunggren et al. 2012; McLean et al. 2008). In addition to V2a interneurons, *vglut2a* is expressed in many other cell types (Higashijima et al. 2004), including V3 interneurons that shape excitatory drive during walking in mice (Zhang et al. 2008) and mechanosensory cell types that feed back to the locomotor network (Douglass et al. 2008; Knafo et al., 2017). We do not have a complete mechanistic understanding of how simultaneous activation of all of the *vglut2a*‐expressing cell types produces locomotor activity. Regardless, this approach does provide an opportunity to test the contributions of the various *vglut2a*‐expressing cell types to production of locomotor activity through targeted ablation and removal from the network.

Continuous ChR2‐induced activation of spinal locomotor networks leads to a gradual decrease in peripheral motor nerve burst frequency and number (Hägglund et al. 2010; Wahlstrom‐Helgren et al. 2019). This reduction in activity is likely influenced by ChR2 desensitization, a property of ChR2 during prolonged blue light activation (Higgins et al. 2017; Nagel et al. 2003). Using zebrafish larvae, we found that the robustness of ChR2‐induced locomotor activity was restored after an interval of rest (Wahlstrom‐Helgren et al. 2019). However, it is not known how well, or if, ChR2‐induced locomotor activity is maintained during repetitive blue‐ light stimulation over long time periods; a requirement of many experimental designs.

Continuous optogenetic stimulation of *vglut2a*‐expressing neurons in spinal cords of larval zebrafish produces locomotor activity with the hallmark characteristics of swimming; coordination (side‐to‐side alternation and rostrocaudal progression) and organization into discrete episodes of activity (Wahlstrom‐Helgren et al. 2019). The objective of this study was to test the tolerance of this optogenetic activation paradigm to repetitive stimulation. Up to 60 continuous, 10 s‐long stimuli were delivered to preparations at different intervals. Notably, locomotor activity persisted at all stimulus intervals, but was diminished after longer combined times of light exposure (i.e., more frequent stimulation). In comparison, corresponding timepoints of NMDA‐induced swimming did not show a change in the number of peripheral nerve bursts, but bursts did become less well organized into discrete swimming episodes. Finally, we confirmed that repetitive stimulation of *vglut2a*‐expressing neurons is an effective approach for within‐subjects study of neuromodulation of spinal CPGs by demonstrating that application of the neuromodulator dopamine (DA) reversibly depressed ChR2‐induced locomotor activity.

## 2 METHODS

### 2.1 Zebrafish lines and care

The University of Minnesota Institutional Animal Care and Use Committee approved all of the protocols used in this study. Adult zebrafish diet and recirculating system water values were described previously (Wahlstrom‐Helgren et al. 2019). Experimental animals were raised in a lighted (14‐h light:10‐h dark) 28.5°C incubator in water containing 60 µg/ml Instant Ocean (Blacksburg, VA) Sea Salt until 7 days post‐fertilization (dpf). Double‐transgenic *Tg(vglut2a*:*Gal4ff)^nns20^*;*Tg(UAS*:*ChR2(H134R)*‐*mCherry)^umn201^* larvae (4–7 dpf; sexually‐undifferentiated) were used in all experiments. The *Tg(vglut2a*:*Gal4ff)^nns20^* (Satou et al. 2013) and *Tg(UAS*:*ChR2(H134R)*‐*mCherry)^umn201^* (Montgomery et al. 2018; Wahlstrom‐Helgren et al. 2019) lines were described previously.

### 2.2 Electrophysiology

Established procedures for peripheral nerve voltage recordings were used to measure fictive swimming (Masino & Fetcho, 2005). Prior to preparation, larvae were anesthetized with 0.02% Tricaine‐S (Western Chemical, Ferndale, WA) in extracellular saline composed of, in mM: 134 NaCl, 2.9 KCl, 1.2 MgCl_2_, 2.1 CaCl_2_, and 10 HEPES, adjusted to pH 7.8 and 290–300 mosM. Larvae were prepared for recording by pinning them laterally through the notochord to a dissecting dish, removing the skin with forceps to expose the peripheral nerves, and paralyzing them with approximately 25 µM α‐bungarotoxin (Cat. No. 2133; Tocris, Bristol, UK) for 10 min. Preparations were either left intact or spinalized by transecting the spinal cord and dorsal body wall with a razor blade at body segment 2/3. Dissecting dishes were placed on an Olympus (Center Valley, PA) BX51 WI compound microscope, and preparations were perfused with extracellular saline at a rate of 0.4 ml/min in a volume of approximately 1 ml. Glass suction electrodes were positioned on the peripheral nerves at the intermyotomal clefts, and recordings were initiated after 20–30 min of perfusion. Voltage signals were amplified with an Axon Multiclamp 700B amplifier connected to a Digidata 1440A digitizer. Signals were sampled at 10 kHz, band‐pass filtered to 100–1000 Hz, and recorded using pClamp 10 software (Molecular Devices, Sunnyvale, CA).

### 2.3 Induction of fictive swimming

Fictive swimming in intact (non‐spinalized) preparations was spontaneous (i.e., not experimentally evoked). ChR2‐induced fictive swimming was evoked by illuminating spinalized preparations with epifluorescent blue light (11.1 mW cm^−2^ nm^−1^; Wahlstrom‐Helgren et al. 2019). A Model 2100 Isolated Pulse Stimulator (A‐M Systems, Sequim, WA) was used to control an X‐Cite 12‐LED Boost lamp (Excelitas Technologies, Waltham, MA). Ten s‐long pulses of continuous light with inter‐stimulus periods of 1, 3, or 15 min were delivered through a FITC filter set (part no. 41001; Chroma Technology, Bellows Falls, VT) and 20× water immersion objective (illuminating 10–12 body segments caudal to the transection). For chemical induction of fictive swimming, spinalized preparations were continuously bath‐perfused with 50 µM NMDA (Cat. No. M3262; Sigma‐Aldrich, St. Louis, MO). Spinalized preparations were perfused with extracellular saline for 10 min, NMDA was added to the perfusate, and peripheral nerve recordings were initiated after 20 min in NMDA.

### 2.4 Dopamine treatment

Spinalized preparations were exposed to 10 s blue light with an inter‐stimulus period of 3 min as described above. After 10 min of baseline peripheral nerve recording in extracellular saline, 10 µM DA hydrochloride (Cat. No. H8502; Sigma‐Aldrich) was added to the perfusate for 10 min. DA was then washed from the bath with extracellular saline.

### 2.5 Data analysis

Peripheral nerve voltage recordings were analyzed by an in‐house program written in MATLAB (The MathWorks, Natick, MA) to measure fictive swimming properties as we described previously (Wiggin et al. 2012). Burst duration was defined as the time from onset to offset of each peripheral nerve burst. Fictive swimming episodes were defined as collections of bursts separated by fewer than 150 ms of quiescence. Episode duration was measured from the time of onset of the first burst of an episode until the offset of the final burst of the same episode. Burst frequency is the inverse of burst period, which was defined as the time from onset of one burst to onset of the subsequent burst. Notably, inter‐episode burst periods (i.e., between active swimming episodes) were excluded from burst frequency calculations; only intra‐episode burst periods were calculated. ChR2‐induced fictive swimming activity was analyzed during 10 s blue light stimuli, and NMDA‐induced fictive swimming activity was analyzed in corresponding 10 s timeframes. Fictive swimming episodes that were interrupted at the end of a 10‐s timeframe were excluded from analysis.

Episodic organization (EO) scores were calculated, as described previously (Wiggin et al. 2012), to compare ChR2‐induced with NMDA‐induced fictive swimming, because swimming episodes and their properties became unquantifiable over time in a subset of NMDA‐treated preparations. This was due to a reduction of inter‐episode burst periods that produced nearly continuous peripheral nerve bursting activity. A critical value was calculated to differentiate between shorter intra‐ and longer inter‐episode burst periods by averaging all burst periods and adding two standard deviations. EO scores were defined as the log_10_ ratio of the mean inter‐episode burst period to the mean intra‐episode burst period. Hence, bimodal distributions of burst periods (higher EO scores) indicate bursting activity that is organized into discrete episodes of fictive swimming. Non‐bimodal distributions of burst periods (lower EO scores) indicate bursting activity that is not organized into discrete episodes.

JMP Pro software (SAS Institute Inc., Cary, NC) was used for statistical analyses. Statistical significance was determined with an α of 0.05, and the test used is indicated in text with each comparison. One SD from the mean is indicated by error bars or shaded areas.

## 3 RESULTS

### 3.1 Comparison of fictive swimming paradigms

Voltage recording of peripheral nerve activity is an effective means for recording the neural correlate of swimming in paralyzed zebrafish (fictive swimming; Masino & Fetcho, 2005). In spinalized preparations, spinal locomotor pattern‐generating networks can be activated by bath application of NMDA (Gabriel et al. 2008; McDearmid & Drapeau, 2006) and, more recently, by optogenetic activation of spinal glutamatergic neurons (Ljunggren et al. 2014; Wahlstrom‐Helgren et al. 2019). The properties of fictive swimming produced under these different swimming paradigms have been characterized individually (Masino & Fetcho, 2005; McDearmid & Drapeau, 2006; Wahlstrom‐Helgren et al. 2019; Wiggin et al. 2012), but not systematically compared. Therefore, we compared the properties of fictive swimming produced spontaneously by intact preparations (N = 7), by NMDA bath application in spinalized preparations (N = 11), and by optogenetic activation of *vglut2a*‐expressing neurons in spinalized preparations (N = 10).

**FIGURE 1:**
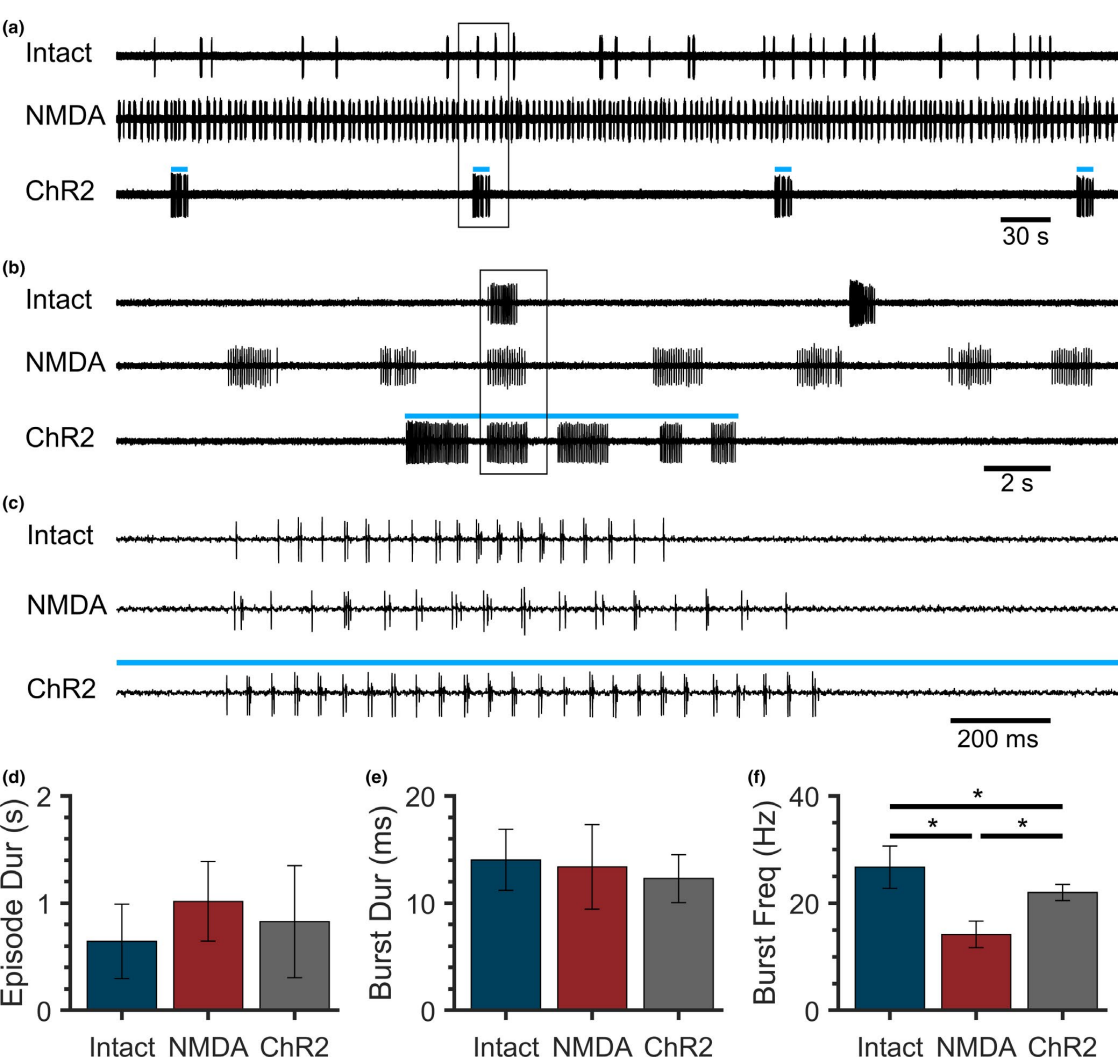
Locomotor properties of intact spontaneous, NMDA‐induced, and ChR2‐induced fictive swimming. (a–c) Fictive swimming activity was recorded from peripheral motor nerves of intact spontaneously swimming (Intact), spinalized NMDA‐induced (NMDA), and spinalized ChR2‐induced (ChR2) *Tg(vglut2a*:*Gal4ff)^nns20^*;*Tg(UAS*:*ChR2(H134R)*‐*mCherry)^umn201^* larval preparations (blue lines represent continuous 10 s blue light stimuli). Peripheral motor nerve voltage traces of 10 min (a), 30 s (b), and 3 s (c) are shown. Boxes indicate regions expanded in subsequent panels. (d–f) Mean values of fictive Episode Duration (d), Burst Duration (e), and Burst Frequency (f). Error bars represent SD. Asterisks indicate statistically significant differences between groups.

Both NMDA‐ and ChR2‐induced fictive swimming properties are influenced by stimulus strength (Wahlstrom‐Helgren et al. 2019; Wiggin et al. 2012); hence NMDA and blue light stimuli were applied at established values of 50 µM (Lambert et al. 2012; Montgomery et al. 2018) and 11.1 mW cm^−2^ nm^−1^ for 10 s (Wahlstrom‐Helgren et al. 2019), respectively. Over a 10 min recording period (Figure 1a), intact preparations (Intact) produced episodes of fictive swimming at variable intervals, spinalized preparations in NMDA (NMDA) produced continuous episodic swimming, and spinalized preparations stimulated with blue light (ChR2) produced swimming activity only when exposed to a blue light stimulus (blue lines). All three swimming paradigms revealed discrete episodes of fictive swimming activity (Figure 1b) that were comprised of similar peripheral nerve bursting patterns (Figure 1c).

Comparisons between fictive swimming paradigms revealed no significant differences in episode duration (Figure 1d; single‐factor ANOVA, *F* = 1.821, *p* = 0.182) or burst duration (Figure 1e; single factor ANOVA, *F* = 0.715, *p* = 0.499). There was a significant effect of swimming paradigm on burst frequency, the neural correlate of tail‐beat frequency (Figure 1f; single factor ANOVA, *F* = 52.280, *p* < 0.001). Burst frequency in intact, spontaneously swimming preparations was significantly higher than that in spinalized NMDA‐induced and ChR2‐induced preparations (Tukey Test, *p* < 0.001 and *p* = 0.003, respectively). Burst frequency was significantly higher during ChR2‐induced fictive swimming than during NMDA‐induced fictive swimming (Tukey Test, *p* < 0.001). Inter‐episode intervals were not compared, due to the irregularity of swimming episodes in intact, spontaneously swimming preparations.

### 3.2 ChR2‐induced locomotor activity persists during repetitive optogenetic stimulation

**FIGURE 2:**
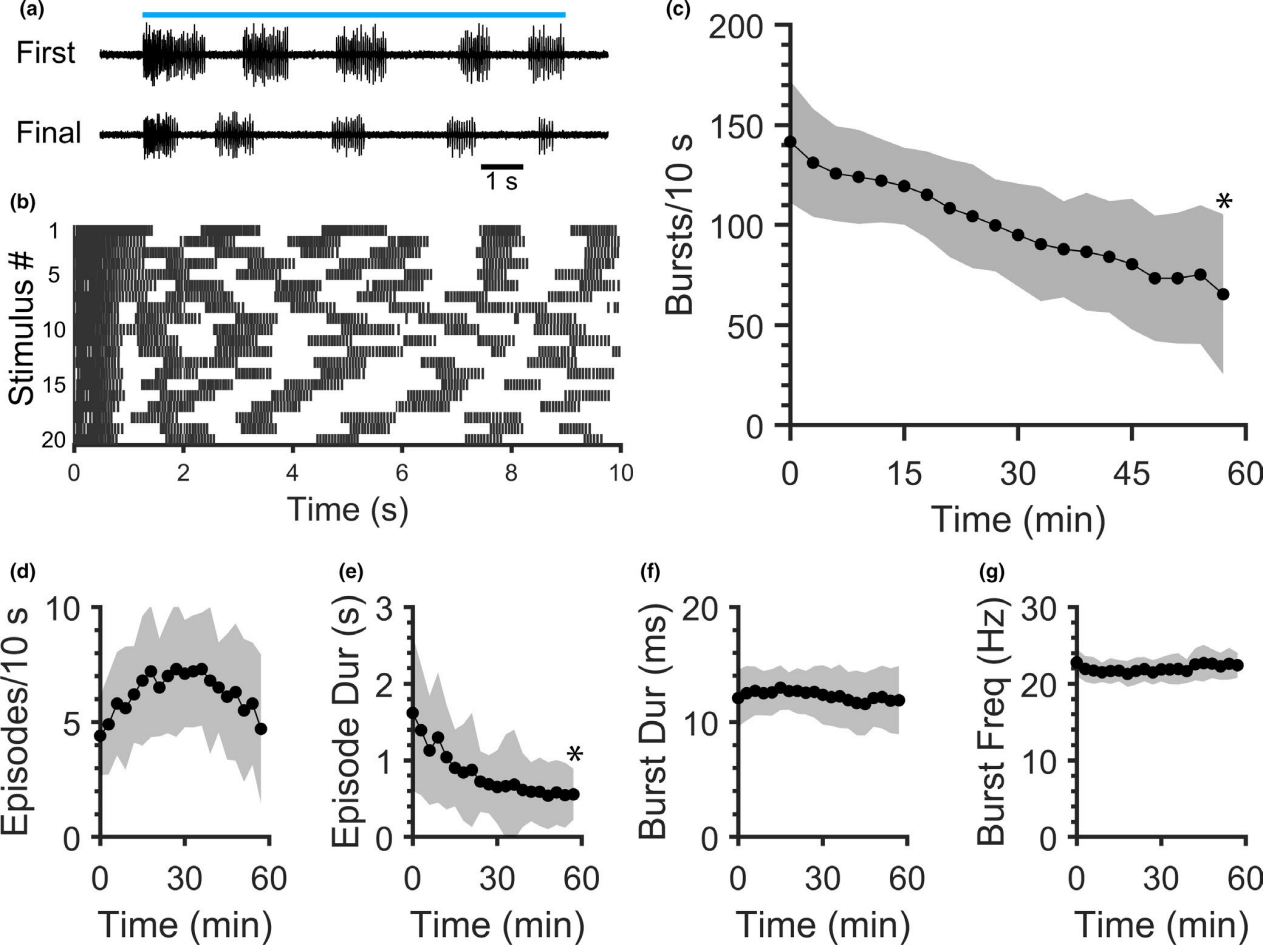
Robustness of ChR2‐induced fictive swimming decreases during repetitive blue light stimulation. Peripheral motor nerve voltage traces were recorded as 20 blue light stimuli were delivered at 3 min intervals. (a) Peripheral nerve voltage traces from a representative preparation during the first (t = 0) and final (t = 57 min) blue light stimuli (blue line represents continuous 10 s blue light stimulus). (b) Raster plot of burst times (vertical lines) for each of the 20 stimuli (rows) delivered to the preparation in (a). (c–g) Mean values of fictive swimming properties during repetitive 10 s stimuli: number of bursts produced during each stimulus (c), number of episodes produced during each stimulus (d), episode duration (e), burst duration (f), and within‐episode burst frequency (g). Shaded regions represent SD. Asterisks indicate significant differences between the first and final (20th) stimuli.

The number of peripheral nerve bursts produced when activating *vglut2a*‐expressing neurons with ChR2 decreases throughout single, sustained (10–60 s) blue light stimuli (Wahlstrom‐Helgren et al. 2019). The effects of repetitive optogenetic stimulation of *vglut2a*‐expressing neurons on ChR2‐induced locomotor activity have not been examined. Therefore, spinalized preparations were exposed to 20 blue light stimuli of 10 s each, with an inter‐stimulus period of 3 min (Figure 2). Representative peripheral nerve traces revealed that episodic fictive swimming activity was produced during the final (20th) stimulus, although there was an apparent reduction, or fatigue, of activity relative to the first stimulus (Figure 2a).

The number of peripheral nerve bursts (the fictive equivalent of tail bends) recorded during each 10 s blue light stimulus was selected as an objective assessment of the quantity of activity produced by the locomotor network. Additional locomotor properties were quantified to describe how repetitive stimulation affected the frequency and organization of this activity. Locomotor fatigue occurred gradually over the 20 stimuli (Figure 2b), characterized by significant reductions in the number of bursts, but not episodes, produced per 10 s stimulus (Figure 2c,d; *t*‐tests, *p* < 0.0001 and *p* = 0.7905, respectively) and mean swimming episode duration (Figure 2e; *t*‐test, *p* = 0.0107), when the final stimulus was compared with the first stimulus (N = 10). Although the number of bursts produced per stimulus decreased, burst properties (mean burst duration and frequency) did not change significantly (Figure 2f,g; *t*‐tests, *p* = 0.7322 and 0.5008, respectively). Hence, fictive swimming persisted when repetitive stimuli were delivered, although the amount of fictive bursting produced during stimuli decreased.

### 3.3 The organization of ChR2‐induced locomotor activity is maintained during repetitive optogenetic stimulation

**FIGURE 3:**
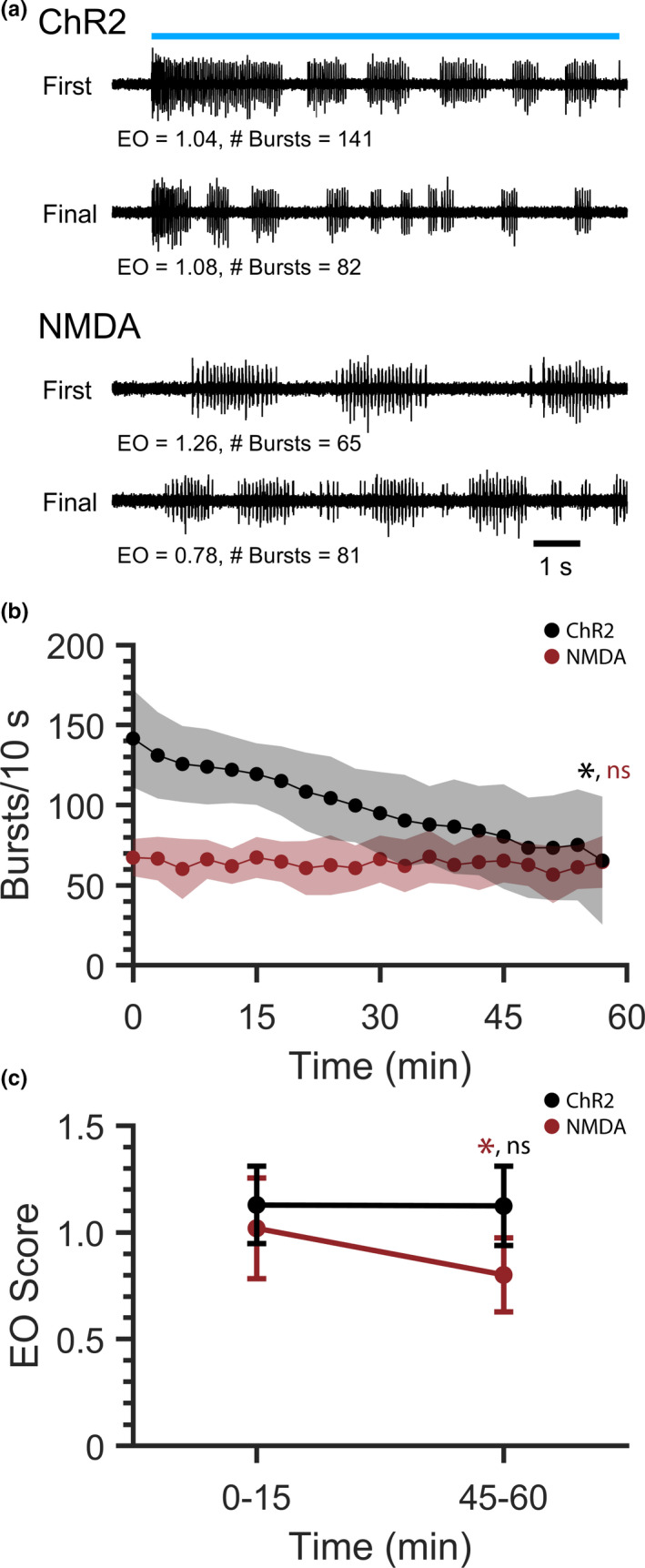
Comparison of robustness and organization of repetitive ChR2‐induced and continuous NMDA‐induced fictive swimming. ChR2‐induced and NMDA‐induced fictive swimming were recorded from spinalized *Tg(vglut2a*:*Gal4ff)^nns20^*;*Tg(UAS*:*ChR2(H134R)*‐*mCherry)^umn201^* preparations. ChR2‐induced swimming was stimulated for 10 s with an inter‐stimulus period of 3 min. Corresponding 10 s‐long windows of NMDA‐induced fictive swimming were analyzed every 3 min. (a) Representative peripheral nerve voltage traces from the first (t = 0) and final (t = 57 min) timepoints of ChR2‐induced (top; blue line represents continuous 10 s blue light stimulus) and NMDA‐induced (bottom) fictive swimming. (b) Total number of bursts detected during each 10 s recording of ChR2‐induced (ChR2; black; plot also shown in Figure 2c) and NMDA‐induced (NMDA; red) fictive swimming. (c) Episodic Organization (EO) scores of ChR2‐induced (black) and NMDA‐induced (red) fictive swimming. Recordings of 10 s were grouped into bins defined by elapsed experimental time. Each bin contained 5 voltage recordings (total of 50 s/bin). Shaded regions and error bars represent SD. Asterisks indicate significant differences when comparing the first and final stimuli in b and bins in c; ns indicates that differences were not significant.

We next tested whether the reduction of peripheral nerve bursting affected the organization of the bursts into discrete episodes of fictive swimming. As NMDA treatment is a conventional approach for activating spinal locomotor networks, optogenetic stimulus timepoints were compared with corresponding 10 s windows of fictive swimming activity induced by continuous application of NMDA. Double‐transgenic *Tg(vglut2a*:*Gal4ff)^nns20^*;*Tg(UAS*:*ChR2(H134R)*‐*mCherry)^umn201^* larvae were spinalized and either exposed to 20 blue light stimuli with an inter‐stimulus period of 3 min (N = 10) or were continuously perfused with 50 µM NMDA (N = 11; Figure 3a). In contrast to the gradual decrease in the number of bursts produced over the course of repetitive optogenetic stimulation (Figure 2c), the number of bursts did not change significantly during NMDA‐induced fictive swimming when comparing the first and final timepoints (Figure 3b, red; *t*‐test, *p* = 0.6039). Fictive swimming activity produced during the first and final five stimuli (i.e., stimuli 1–5 and 16–20) was concatenated, and episodic organization (EO) scores were calculated (Wiggin et al. 2012) to determine how well peripheral nerve bursts were grouped into discrete episodes of fictive swimming (Figure 3c). EO score did not change during repetitive optogenetic stimulation (*t*‐test, *p* = 0.6039), but did decrease significantly during NMDA‐induced fictive swimming (*t*‐test, *p* = 0.0033). Altogether, fictive swimming activity induced by repetitive optogenetic stimulation remained episodically organized, despite a decrease in the number of bursts produced. By contrast, NMDA‐induced fictive swimming exhibited no change in burst number, but became less organized over time.

### 3.4 DA depresses ChR2‐induced locomotor activity

**FIGURE 4:**
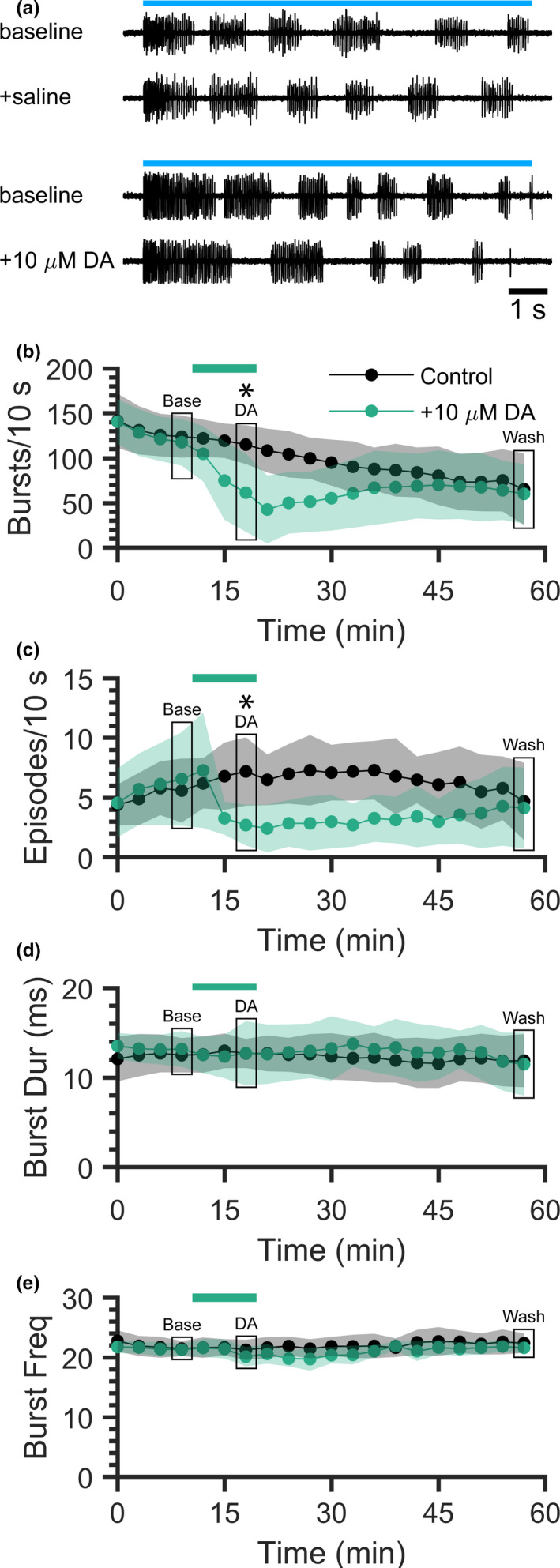
Application of exogenous dopamine reduces ChR2‐induced fictive swimming activity. Fictive swimming was induced in spinalized *Tg(vglut2a*:*Gal4ff)^nns20^*;*Tg(UAS*:*ChR2(H134R)*‐*mCherry)^umn201^* preparations by delivering 10 s‐long blue light stimuli at 3 min intervals. Dopamine (DA) treatment preparations were perfused with extracellular saline for 10 min, 10 µM DA was added to the perfusate for 10 min, followed by wash out of DA with saline. Control preparations were continuously perfused with extracellular saline. (a) Fictive swimming activity of an untreated control preparation (top) at t = 0 (baseline) and t = 18 min (+saline) and of a DA‐treatment preparation (bottom) at t = 0 (baseline) and t = 18 min (+10 µM DA). Blue lines represent continuous 10 s blue light stimuli. (b–e) Number of bursts produced during each 10 s stimulus (b), number of swimming episodes produced during each 10 s stimulus (c), mean burst duration (d), and mean burst frequency (e) of DA‐treatment (green) and untreated control (black) preparations. Horizontal green bar indicates time of 10 µM DA application. Shaded regions represent SD. Boxes indicate final baseline (Base), DA treatment (DA), and washout (Wash) stimuli that were compared between control (plots also shown in Figure 2) and DA‐treatment groups. Asterisks indicate significant differences between groups.

The sole source of spinal DA is a population of diencephalic neurons that project to the spinal cord via the dopaminergic diencephalospinal tract (Tay et al. 2011). These neurons also project to several regions of the brain, making it challenging to examine the influence of DA specifically on the spinal locomotor network. To determine if repetitive optogenetic stimulation might be effectively used to study the effects of neuromodulators like DA on the spinal locomotor network, exogenous DA was applied to preparations during the repetitive stimulation paradigm (Figure 4). Blue light stimuli were delivered to spinalized preparations every 3 min, and DA (10 µM) was added to the perfusate following the 4th baseline stimulus (Figure 4a). DA was removed by perfusing preparations with DA‐free saline following the seventh stimulus. Fictive swimming properties during the final baseline (4th, Base), final DA treatment (7th, DA), and final washout (20th, Wash) stimuli were compared between the DA‐treatment (N = 7) and untreated control (N = 10) groups (Figure 4b–e).

We confirmed that there were no differences in fictive swimming properties during the Base stimulus between DA‐treatment and untreated control preparations (*t*‐tests): bursts per stimulus (117.7, SD 26.1 bursts and 124.0, SD 23.5 bursts, respectively; *p* = 0.6206), episodes per stimulus (6.6, SD 3.9 episodes and 5.6, SD 2.7 episodes, respectively; *p* = 0.5811), burst duration (13.2, SD 2.0 ms and 12.5, SD 1.9 ms, respectively; *p* = 0.4756), and burst frequency (21.3, SD 1.0 Hz and 21.5, SD 1.3 Hz, respectively; *p* = 0.7311). Fewer bursts were produced during the final DA treatment stimulus in DA‐treatment preparations (61.4, SD 43.6 bursts) than in untreated control preparations (115.1, SD 21.6 bursts; *t*‐test, *p* = 0.0166), and there was no difference during the Wash stimulus between treated (60.1, SD 33.2 bursts) and untreated preparations (Figure 4b; 65.4, SD 40.0 bursts; *t*‐test, *p* = 0.7722). Fewer fictive swimming episodes were produced during the final DA treatment stimulus in DA‐treatment preparations (2.7, SD 1.7 episodes) than in untreated preparations (7.2, SD 2.9 episodes; *t*‐test, *p* = 0.0011), and there was no difference during the Wash stimulus between treated (4.1, SD 3.4 episodes) and untreated preparations (Figure 4c; 4.7, SD 3.2 episodes; *t*‐test, *p* = 0.7394). There were no differences in burst duration between treatment and control groups during the final DA treatment stimulus (12.7, SD 3.6 ms and 12.7, SD 1.6 ms, respectively; *t*‐test, *p* = 0.9947) or during the Wash stimulus (Figure 4d; 11.5, SD 3.5 ms and 11.9, SD 3.0 ms, respectively; *t*‐test, *p* = 0.8124). There were also no differences in burst frequency between DA‐treatment and untreated preparations during the final DA treatment stimulus (20.1, SD 1.6 Hz and 21.3, SD 1.7 Hz, respectively; *t*‐test, *p* = 0.1873) or the Wash stimulus (Figure 4e; 20.6, SD 1.6 Hz and 21.7, SD 1.8 Hz, respectively; *t*‐test, *p* = 0.2241). Overall, DA treatment reversibly decreased ChR2‐induced locomotor activity (number of bursts and episodes) without affecting burst duration or frequency.

### 3.5 Effects of inter‐stimulus interval on locomotor fatigue

**FIGURE 5:**
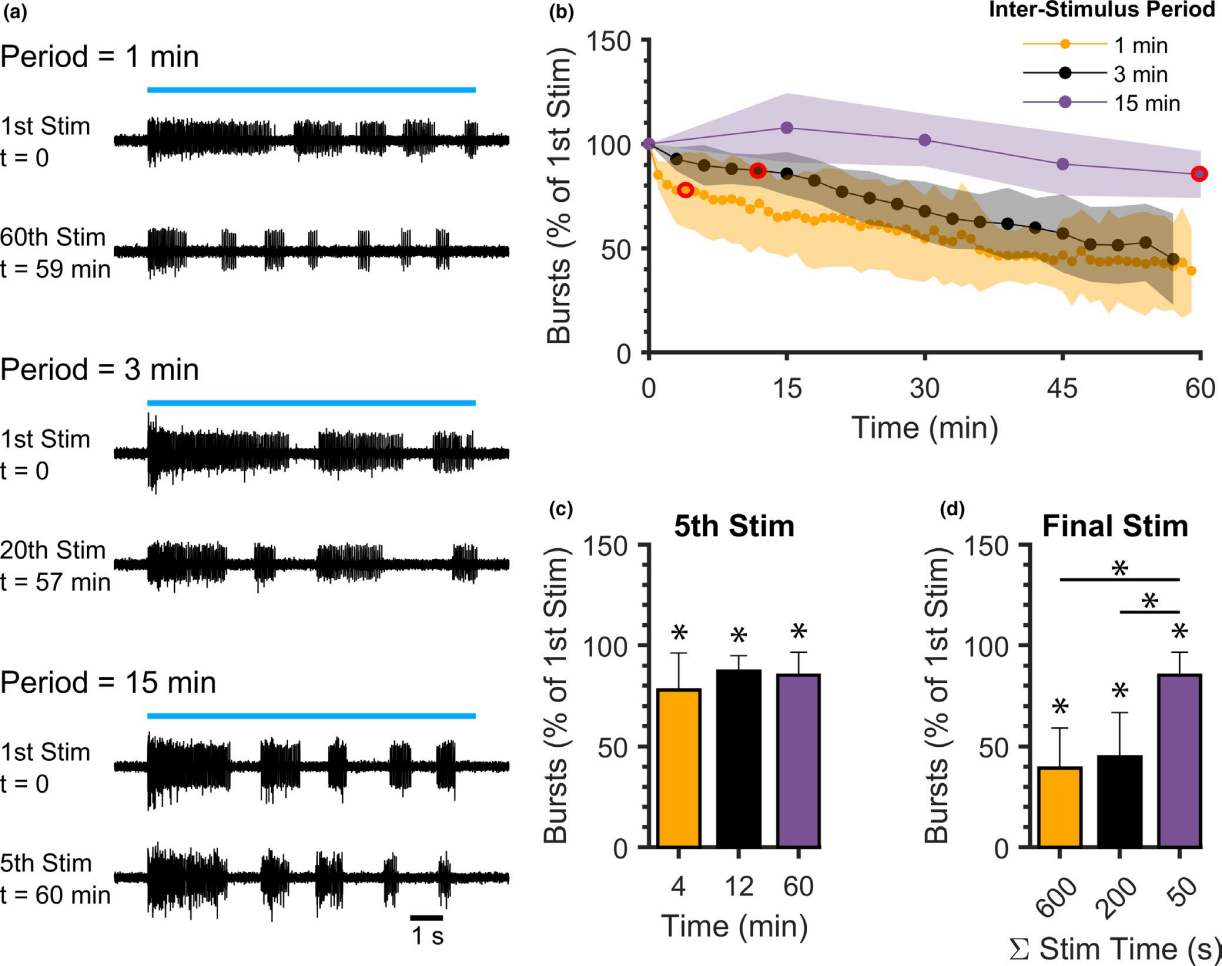
Effects of repetitive blue light stimulus exposure times and frequencies on fatigue of fictive locomotor bursting activity. Ten s‐long blue light stimuli were delivered to spinalized *Tg(vglut2a*:*Gal4ff)^nns20^*;*Tg(UAS*:*ChR2(H134R)*‐*mCherry)^umn201^* preparations with inter‐stimulus periods of 1 (60 stimuli over 59 min), 3 (20 stimuli over 57 min), and 15 min (5 stimuli over 60 min). (a) Representative peripheral nerve recordings of fictive swimming activity produced during the first (t = 0) and final 10 s blue light stimulus delivered with each inter‐stimulus period (blue lines represent continuous 10 s blue light stimuli). (b) Number of bursts (normalized to the first stimulus at t = 0) produced during 10 s blue light stimuli delivered with each inter‐stimulus period. Red circles indicate the fifth stimulus at each inter‐stimulus period. (c) Number of bursts (normalized to the first stimulus at t = 0) produced during the fifth blue light stimulus (red circles in b) for each inter‐stimulus period (expressed as time of fifth stimulus delivery). (d) Number of bursts (normalized to the first stimulus at t = 0) produced during the final blue light stimulus, expressed as the total amount of time stimulated with blue light for each inter‐stimulus period. Shaded regions and error bars represent SD. Asterisks indicate statistically significant differences from the first stimulus. Asterisks over horizontal lines indicate significant differences between groups.

To determine if varying stimulus number and frequency would affect the fatigue of fictive swimming activity during repetitive optogenetic activation, preparations were exposed to 10 s stimuli 60 times with an inter‐stimulus period of 1 min (N = 7), 20 times with an inter‐stimulus period of 3 min (N = 10), or 5 times with an inter‐stimulus period of 15 min (N = 7; Figure 5). Episodic fictive swimming activity was maintained through the final stimulus at each stimulation frequency (Figure 5a). We confirmed that the first stimulus of the 1 min (127.4, SD 36.0 bursts), 3 min (141.6, 30.4 SD bursts), and 15 min (116.9, 25.9 SD bursts) period groups did not produce significantly different numbers of bursts (single‐factor ANOVA, *F* = 1.3560, *p* = 0.2793). As groups did not show an initial difference, the number of bursts produced was normalized to the first stimulus for all groups and stimuli for comparison (Figure 5b).

To test the influence of experiment duration (i.e., time elapsed since spinal transection or first blue light application) on fatigue of optogenetically induced fictive swimming, the number of bursts produced during the fifth stimulus of each group were compared (Figure 5c). The fifth stimulus was delivered at different experimental timepoints in each group (4 min with 1 min period, 12 min with 3 min period, and 60 min with 15 min period; Figure 5b, red circles). Fewer bursts were produced during the fifth stimulus than during the initial stimulus for all groups: 1 min period (78.0% of first stimulus, SD 18.3%; *t*‐test, *p* = 0.0188), 3 min period (87.2% of first stimulus, SD 7.7%; *t*‐test, *p* = 0.0005), and 15 min period (85.3% of first stimulus, SD 11.2%; *t*‐test, *p* = 0.0135). Notably, no between‐groups differences were observed during the fifth stimulus (single factor ANOVA, *F* = 1.1824, *p* = 0.3261). This indicated that when total stimulus exposure time was equal (50 s), experiment duration and stimulus period did not significantly impact the number of bursts produced.

All stimulus periods exhibited a reduced number of bursts when the final stimulus was compared with the first (Figure 5d). Preparations that were stimulated with a 1‐min period (600 s combined stimulation time) produced 39.2% (SD 20.0%) as many bursts as during the first stimulus (*t*‐test, *p* = 0.0002). Preparations that were stimulated with a 3‐min period (200 s combined stimulus time) produced 44.8% (SD 21.9%) as many bursts as the first stimulus (*t*‐test, *p* < 0.0001) and those stimulated with a 15‐min period (50 s combined stimulus time) produced 85.3% (SD 11.2%) as many bursts as the first stimulus (*t*‐test, *p* = 0.0135). There was a significant effect of inter‐stimulus period on the number of bursts generated during the final stimulus (single‐factor ANOVA, *F* = 13.0051, *p* = 0.0002). Inter‐stimulus periods of 1 and 3 min resulted in a larger reduction in the number of bursts produced than did a period of 15 min (Tukey Test, *p* = 0.0005 and 0.0008, respectively), and 1 and 3 min periods were not significantly different from one another (Tukey Test, *p* = 0.8224). Hence, fatigue of ChR2‐induced fictive swimming activity was mitigated by increasing the inter‐stimulus interval when experiment duration was equivalent (*t* = 57–60 min).

## 4 DISCUSSION

Bath application of NMDA has conventionally been used to activate spinal locomotor networks to induce locomotor activity in spinalized animals; however, the cellular and network mechanisms underlying NMDA‐induced swimming remain unclear. It is understood that NMDA increases a network's excitability state through activation of NMDA receptors (Sharples & Whelan, 2017). It has been proposed that the episodic nature of NMDA‐induced swimming is a product of slow intrinsic motor neuronal membrane oscillations (McDearmid & Drapeau, 2006), which would suggest that NMDA‐induced swimming episodes are not generated by spinal locomotor CPGs. However, we and others have shown that NMDA receptors may not be necessary for expression of organized locomotor activity (Beato et al. 1997; Talpalar & Kiehn, 2010; Wahlstrom‐Helgren et al. 2019). Furthermore, NMDA receptor activity is directly inhibited by activation of certain serotonin receptors (Murase et al. 1990; Yuen et al. 2005), potentially limiting the usefulness of NMDA‐induced locomotion paradigms for the study of serotonergic signaling. Indeed, we found that serotonin reversibly inhibits NMDA‐induced fictive swimming in spinalized larval zebrafish (Montgomery et al. 2018), despite commonly being considered to facilitate spinal generation of locomotor activity following spinal cord transection and injury (Fouad et al. 2010; Ghosh & Pearse, 2014; Jacobs & Fornal, 1993). Hence, it is imperative to develop and characterize novel paradigms of spinal locomotor network activation to examine their components and modulation.

### 4.1 Comparison of properties of fictive swimming paradigms

We directly compared the properties of spontaneous swimming in intact larval preparations to swimming evoked by ChR2 and NMDA in spinalized preparations. Fictive swimming occurred spontaneously in intact preparations, whereas fictive swimming occurred continuously in NMDA bath‐perfused spinalized preparations and occurred only during blue light illumination in optogenetically‐induced spinalized preparations (Figure 1a,b). Although we did not find a significant difference in episode duration between swimming paradigms, trends were in agreement with previous work that found that NMDA‐induced swimming episode durations are longer than intact spontaneous episode durations (Lambert et al. 2012; Wiggin et al. 2012). As ChR2‐induced episode durations decreased during repetitive optogenetic stimulation (Figure 2e), these comparisons are affected by the number of stimuli that preparations were exposed to. We also did not find any differences in burst duration between the swimming paradigms (Figure 1e). Burst duration was reported to be shorter during NMDA‐induced swimming in spinalized preparations than in spontaneously‐swimming intact preparations; however, this difference is dependent on rostrocaudal recording location (Wiggin et al. 2012), which was not tested in the present study. We and others have reported that burst frequency is higher during spontaneous swimming in intact zebrafish larvae (Buss & Drapeau, 2001; Masino & Fetcho, 2005) than during NMDA‐induced swimming in spinalized preparations (McDearmid & Drapeau, 2006; Wiggin et al. 2012). The present study corroborated these findings and additionally found that ChR2‐induced burst frequency was both significantly slower than intact swimming and faster than NMDA‐induced swimming (Figure 1f). Altogether, this suggests that the properties of swimming elicited through optogenetic activation of *vglut2a*‐expressing spinal neurons are more similar to endogenously‐produced swimming than swimming elicited through application of exogenous NMDA.

### 4.2 Properties of locomotor activity during repetitive optogenetic stimulation

When using acute pharmacological treatments in repeated‐measures experimental designs, it is imperative that locomotor activity be induced over a prolonged time so that activity can be recorded during baseline, treatment, and treatment‐free wash‐out conditions. Although NMDA‐induced locomotor activity maintained its robustness over a 1 h‐long timecourse (Figure 3b), we observed a degradation in the organization of swimming activity, sometimes to the point that bursting became tonic and did not match our definition of swimming. This effect was quantified as a reduction in EO score over the timecourse (Figure 3c).

Continuous (5–60 s) optogenetic activation of spinal glutamatergic neurons elicits coordinated and organized locomotor activity in spinalized zebrafish larvae (Ljunggren et al. 2014; Wahlstrom‐Helgren et al. 2019). Therefore, we developed and tested a repetitive activation paradigm in which twenty 10 s‐long continuous blue light stimuli were applied to spinalized larvae at 3 min intervals. This stimulation paradigm was robust, as preparations consistently generated organized fictive swimming activity throughout the repetitive stimulation timecourse (Figure 3c). However, the fictive swimming response to each blue light stimulus diminished over successive presentations of the stimulus. Although burst frequency (the neural correlate of tail beat frequency) and duration did not change over the timecourse, the number of bursts produced per stimulus decreased, which corresponded to changes to the episodic properties of swimming (Figure 2).

Increasing the number of stimuli from 20 to 60 by reducing the inter‐stimulus period from 3 to 1 min did not reduce the number of bursts produced during repetitive stimuli over a similar experimental timespan (i.e., 60 min; Figure 5). However, decreasing the number of stimuli to five by increasing the inter‐stimulus period to 15 min partially mitigated this locomotor fatigue; the number of bursts per stimulus decreased, but this reduction was smaller than at the other stimulus intervals (Figure 5). This suggested that the total time of blue light exposure influenced the maintenance of locomotor robustness, although we were unable discriminate this effect from the potential influence of stimulus interval, which would require varying the frequency and duration of blue light stimuli within trials.

This inter‐stimulus locomotor fatigue differed from our previous description of diminished activity within a single, continuous stimulus, in which the number of bursts produced in the first half of a stimulus was greater than the number of bursts produced during the second half of the stimulus (Wahlstrom‐Helgren et al. 2019). Even so, similar mechanisms may underlie both effects. First, all ChR2 variants exhibit some degree of desensitization during prolonged activation (Higgins et al. 2017; Nagel et al. 2003; Zamani et al. 2017). Increased inter‐stimulus periods may assist recovery from desensitization. Additionally, long‐term postsynaptic depression may have occurred through glutamate receptor desensitization. NMDA receptors are more resistant to desensitization than other glutamate receptors (Trussell & Fischbach, 1989), which is congruent with our observations that NMDA‐induced locomotor activity did not exhibit a reduction in the number of bursts (Figure 3b) and that ChR2‐induced locomotor activity is more dependent on AMPA receptors than NMDA receptors (Wahlstrom‐Helgren et al. 2019). Finally, it is possible that the condition of the spinalized preparations deteriorated over the 1 h‐long experimental timecourse. Although there is no evidence of deteriorated activity in intact (i.e., non‐spinalized) preparations, decreased tissue health resulting from spinalization could manifest as reduced bursting activity during repetitive optogenetic stimulation and as reduced organization during continuous NMDA application. Such an effect would likely result from exposure of the spinal cord to the external environment (i.e., extracellular saline) and/or necrosis near the site of injury.

### 4.3 Repetitive optogenetic stimulation to test effects of DA on fictive swimming

Locomotor fatigue resulting from repetitive optogenetic stimulation could confound repeated‐measures experiments by masking experimental effects on locomotor output. Therefore, we tested the efficacy of the repetitive stimulation paradigm for these studies by treating preparations with exogenous DA. DA is a potent modulator of spinal locomotor networks that exerts concentration‐dependent effects by acting through higher‐affinity D2‐like receptors to inhibit activity at low DA concentrations and through lower‐affinity D1‐like receptors to facilitate activity at high DA concentrations (Clemens et al. 2012; McPherson & Kemnitz, 1994; Svensson et al. 2003).

Methodological differences in earlier studies have prevented a consensus on the effects of spinal dopaminergic signaling on zebrafish locomotor activity. Application of exogenous DA reduces the number of spontaneous swimming episodes in intact 3 and 5 dpf larvae, but not the number of NMDA‐induced episodes in spinalized 3 dpf larvae (Thirumalai & Cline, 2008). Therefore, the reduced initiation of swimming was hypothesized to arise from dopaminergic signaling within the brain and not the spinal cord (Thirumalai & Cline, 2008). The same study also found no effect of DA on other NMDA‐induced swimming parameters, including episode duration. By contrast, both spontaneous (intact) and NMDA‐induced (spinalized) swimming episode durations were reduced in 4–7 dpf larvae (Lambert et al. 2012). This would indicate that dopaminergic signaling within the spinal cord is at least partially responsible for inhibiting locomotor activity between 4 and 7 days of development. These differences may have an ontogenic origin, because aminergic innervation of the spinal cord is not considered morphologically or functionally mature until 4 dpf (Buss & Drapeau, 2001; Lambert et al. 2012; McLean & Fetcho, 2004; Montgomery et al. 2016; Thirumalai & Cline, 2008). Furthermore, network excitability state, when established by varying NMDA concentration, determines the degree to which DA modulates spinal locomotor network activity in lamprey and neonatal mice (Sharples & Whelan, 2017; Svensson et al. 2003). This may also help to reconcile the reported differences in spinalized zebrafish larvae, because these studies used different concentrations of NMDA to induce swimming (Lambert et al. 2012; Thirumalai & Cline, 2008).

Our repetitive optogenetic stimulation paradigm, in which fictive swimming activity was induced by activating endogenous sources of glutamate, similarly revealed a depressive effect of exogenous DA on spinally generated locomotor activity. DA treatment reversibly decreased the number of bursts and episodes produced in spinalized 4–7 dpf zebrafish larvae without affecting burst frequency or duration (Figure 4). This demonstrated that when appropriate controls are used to account for locomotor fatigue, repetitive optogenetic stimulation of *vglut2a*‐expressing neurons can be used effectively in longer‐term (tested up to 1 h) experimentation on spinal locomotor networks.

## 5 CONCLUSION

We previously described an approach to optogenetically activate ChR2‐expressing glutamatergic neurons to generate coordinated and episodic swimming in spinalized zebrafish larvae through continuous application of blue light (Wahlstrom‐Helgren et al. 2019). This synaptic release of endogenous glutamate to target its native receptors is an arguably more biologically relevant method of activating spinal locomotor CPGs than bath‐application of exogenous NMDA. Here, we explored the use of this technique in longer‐duration experimental conditions by applying multiple 10 s blue light stimuli for up to 1 h. This system was robust enough to produce fictive locomotor activity after 60 stimuli; however, there was a significant reduction in activity (i.e., number of bursts) at all stimulus intervals tested (Figure 5). Despite this limitation, we demonstrated that the repetitive optogenetic stimulation paradigm can be effectively used to investigate modulation of locomotor networks (Figure 4) and that locomotor fatigue was partially mitigated by increasing the inter‐stimulus interval to 15 min (Figure 5). We anticipate that development of additional ChR2 variants that are more resistant to desensitization will further alleviate locomotor fatigue arising from repetitive stimulation.

## CONFLICTS OF INTEREST

The authors have no conflicts of interest to report.

## AUTHORS’ CONTRIBUTIONS

JM, MM, and SW‐H conceived the project and designed experiments. JM, KV, and SW‐H performed experiments. JM and SW‐H analyzed data and prepared figures. JM drafted the manuscript. All authors revised the manuscript and approved the final version.
